# Randomised trials in context: practical problems and social aspects of evidence-based medicine and policy

**DOI:** 10.1186/s13063-015-0917-5

**Published:** 2015-09-01

**Authors:** Warren Pearce, Sujatha Raman, Andrew Turner

**Affiliations:** Institute for Science and Society, School of Sociology and Social Policy, University of Nottingham, University Park, Nottingham, NG7 2RD UK; Data to Knowledge Research Group, School of Social and Community Medicine, Oakfield House, University of Bristol, Oakfield Grove, Clifton, BS8 2BN UK

## Abstract

Randomised trials can provide excellent evidence of treatment benefit in medicine. Over the last 50 years, they have been cemented in the regulatory requirements for the approval of new treatments. Randomised trials make up a large and seemingly high-quality proportion of the medical evidence-base. However, it has also been acknowledged that a distorted evidence-base places a severe limitation on the practice of evidence-based medicine (EBM). We describe four important ways in which the evidence from randomised trials is limited or partial: the problem of applying results, the problem of bias in the conduct of randomised trials, the problem of conducting the wrong trials and the problem of conducting the right trials the wrong way. These problems are not intrinsic to the method of randomised trials or the EBM philosophy of evidence; nevertheless, they are genuine problems that undermine the evidence that randomised trials provide for decision-making and therefore undermine EBM in practice. Finally, we discuss the social dimensions of these problems and how they highlight the indispensable role of judgement when generating and using evidence for medicine. This is the paradox of randomised trial evidence: the trials open up expert judgment to scrutiny, but this scrutiny in turn requires further expertise.

## Background

Randomised trials can provide excellent evidence of treatment benefit in medicine. In the last century they have become cemented in the regulatory requirements for the approval of new treatments [1, 2]. Conducting trials and synthesising evidence from trials have themselves become specialised industries. Furthermore, the method of random assignment to control versus test group has attracted renewed attention in the world of public and social policy where it originated in the early 20^th^ century in psychology experiments in education [3]. Randomised trials make up a large and seemingly high-quality proportion of the medical evidence-base.

Evidence-based medicine (EBM) is ‘the conscientious, explicit and judicious use of current best evidence in making decisions about the care of individual patients’ [4]. Over the last twenty years, social scientists studying the EBM movement have stressed that because there is no algorithmic way to practice EBM, the use of clinical expertise to interpret and integrate research evidence with patient values is always contingent on social and political factors. To take two examples, much excellent work has been conducted at the micro-level, looking at guideline development for instance, [5–8], and at the macro-level, looking at the politics of EBM [9–13].

One crucial point that has been increasingly acknowledged, however, is the severe limitation that a distorted evidence-base places on the practice of EBM [14–18]. We examine this in three different contexts: the clinical setting, regulatory decision-making on drug approvals, and health policymaking, where decisions on approved interventions (for example, for health screening) are made drawing on evidence from randomised trials (and that clinicians are then supposed to follow). Due to limitations of space, we do not delve into the separate question of how complex interventions for promoting health outcomes (for example, to reduce smoking or obesity) should be evaluated, that is, whether randomisation is appropriate or even feasible in such cases.

We proceed as follows. First, we describe four important ways in which the evidence from randomised trials is limited or partial: the problem of applying results, the problem of bias in the conduct of randomised trials, the problem of conducting the wrong trials and the problem of conducting the right trials the wrong way. These problems are not intrinsic to the method of randomised trials or the EBM philosophy of evidence; nevertheless they are genuine problems that undermine the evidence that randomised trials provide for decision-making and therefore undermine EBM in practice. Finally, we discuss the social dimensions of these problems and how they highlight the indispensable role of judgement when generating and using evidence for medicine.

## Review

### The problem of applying results from randomised trials

The average result from a study (or more likely, the average result from many pooled studies) may not apply to a target population. The problem of working out when results can be applied is often called the problem of external validity [19], or the problem of extrapolation [20]. Randomised trials have poor external validity because they are designed to provide good evidence that the treatment really is having an effect within the study population.

Philosopher of science, Nancy Cartwright, has clarified the problem of applying randomised trial results, both in medicine [21–23] and in policy [24]. Cartwright tells us that from successful randomised trials we can gain good evidence that the treatment had a positive effect on the outcome in question in some of the study participants. If we are worried about the external validity of randomised trials, it is because what we want is evidence for a different claim, namely, whether the treatment will be effective in some individuals in a target population. (We can be more or less stringent about what effective means here; perhaps just that the treatment helps some even though it may harm others or that it is mostly useless in all but a few.) According to Cartwright, this claim is not supported by the evidence we gain from randomised trials. Further evidence must be provided. The problem of external validity therefore is not finding out what the results from randomised trials tell us about treatment effects in target populations: on their own, randomised trials are poor evidence for that. Rather the problem is finding the *additional* evidence that is needed to apply results from randomised trials to *other* populations. For example, additional evidence exists for whether *this* patient will likely benefit, or how a prevalent comorbidity will affect the treatment effect.

The problem posed by external validity, especially as formulated by Cartwright, highlights the other evidential work that needs to be done to apply the results from randomised trials. Depending on our knowledge about study and target populations, however, this evidence may be more or less straightforward to come by. First, for example, if we have many randomised trials in heterogeneous populations showing a consistent effect, we have some evidence for the robustness of a treatment's effect. Secondly, there are also well-known barriers: we know to be cautious about applying results from drug trials in adults to pediatric populations because we know that children and neonates do not typically behave like 'little adults' in matters of drug absorption, distribution, and metabolism.^1^

Cartwright claims that the other evidence that is required for applying the results of trials is often de-emphasised or ignored. In comparison to existing tools for assessing whether randomised trials provide good evidence that the treatment was effective in the study population, there are few accounts of what the other evidence is or when it counts as good evidence [22]. Furthermore attending to the other evidence that is needed alongside randomised trial evidence, according to Cartwright, is beneficial because clarity about what is needed focuses attention on the details and dynamics that will affect the treatment affect in the target populations, rather than on the confused, demanding and wasteful request for 'similarity' between populations [24].

In response to Cartwright, Petticrew and Chalmers [25] ask what assumptions are legitimate to make about the evidence needed to apply results from randomised trials. Other evidence may be needed, but as a matter of fact, it may also be readily available. They suggest conceptualising the problem of external validity ‘the other way round’, echoing a suggestion made by Rothwell [26] that: ‘The results of trials should be assumed to be externally valid unless there are specific reasons to put this assumption into significant doubt’. Either way round, expert subject knowledge is required to make judgements about external validity. In fact, a subsequent point made by Rothwell is perhaps the most salient, namely, that the description of trials must be sufficiently detailed to permit one to judge what other evidence is needed and where to look for it [26].

### The problem of bias in the conduct of randomised trials

There have been a series of systematic reviews over the last 10 years [27–30] demonstrating that industry-funded trials are more likely to have pro-funder results and conclusions. Findings reported in the results section of trials are more likely to favour the funder (their treatment is more effective or less harmful than the comparator), and the way this gets written into the conclusions also favours the funder (by playing up or playing down particular results).

Some examples of specific studies that have looked at this phenomenon are herein provided. Bourgeois, Murthy and Mandl [31] examined 546 registered trials of five different classes of drug, finding that 85 % of those with an industry sponsor had a favourable outcome; 50 % of those with a government sponsor had a favourable outcome; and 72 % of those with a non-profit sponsor had a favourable outcome. Of those with a non-profit sponsor, however, those with an industry contribution had favourable outcomes in 85 % of cases, compared to 62 % of those without an industry contribution. Djulbegovic *et al.* [32] examined 136 trials of treatments for multiple myeloma, finding that in trials with a non-profit sponsor, the new therapy was reported as better than standard treatment in 53 % of cases, whereas in trials with a for-profit sponsor, this was 74 %. Fries and Krishnan [33] looked at 45 abstracts of industry sponsored randomised trials from the American College of Rheumatology meetings and found that 100 % of the trials favoured the sponsor's drug. Many other similar studies, over the course of 20 years, have found this asymmetry between the results of trials funded by industry and by other sources [34, 35]. Nevertheless, it is important not to overgeneralise the tempting narrative of industry bias, as illustrated by the case of statin trials [36].

Along with the observation that industry-funded trials are more likely to have favourable results for the funder's treatment, many of the studies and systematic reviews above note that industry-funded trials are of equal or higher quality than non-industry funded trials. They rank at least as well on risk of bias measures. That is to say, industry-funded trials are not systematically worse at adequately blinding participants or using proper allocation methods and concealment, and so on. Consequently authors have outlined a range of potential mechanisms that are not typically captured in risk-of-bias assessment tools, by which industry interests can influence study results [37].

Such mechanisms include the strategic design, analysis and reporting of trials [38]. To give some examples, in the design of trials, comparators can be chosen to test a new treatment against the current best treatment at the wrong dose, for the wrong duration, or using something other than the current best treatment as the comparator. Also, outcome measures can be chosen that exaggerate the effect. Charman *et al.* [39] found at least 13 'named' scales for atopic eczema, many scales that were modified versions of existing scales, and others that were newly invented or unpublished (Unpublished scales are particularly dangerous, because they can be constructed post hoc [40]). In the analysis of trial results, interests can be promoted by finding subgroups that show a desirable and significant effect. Star signs are a favourite way to demonstrate the problem. For example, in the ISIS-1 trial, the benefit of the intervention was four times greater in Scorpios [41], and in the ISIS-2 trial, Geminis and Libras did slightly worse when they got the intervention [42]. Equally in the reporting of trial results, interests can influence the way particular results are emphasised or framed, notably, by choosing to use relative rather than absolute measures (20 % relative improvement rather than 5 % or 6 %) [43]. This influence also works by having multiple primary outcomes, or reporting the insignificant ones as secondary outcomes, and even introducing significant results as new primary outcomes [44, 45]. Furthermore, meta-analyses, just like individual studies, suffer from these reporting biases. Jørgensen *et al*. [46] looked at industry-funded and Cochrane meta-analyses of the same drugs. None of the Cochrane reviews recommended the drug in their conclusion, whereas all of the industry-funded reviews did.

In addition to these internal mechanisms affecting design, analysis and reporting, there are also external mechanisms for influencing the total evidence base. The most obvious is publication bias. For example, the multiple publication of positive studies becomes a problem when it is 'covert' and leads to double-counting in meta-analyses. Tramer *et al*. [47] examined 84 published trials of ondansetron for postoperative emesis, which in total contained data on 20,181 patients, of which 11,980 received the treatment. They found that 17 % of trials duplicated data, and that 28 % of the data on the 11980 patients given ondansetron was duplicated. Furthermore in the subgroup of 19 trials that compared prophylactic ondansetron against placebo, three of these trials were duplicated into six further publications. Importantly, meta-analysis comparing the duplicated set of 25 trials against the set of 19 originals showed that duplication led to a 23 % overestimate of the number needed to treat.

As an alternative to covertly publishing positive studies multiple times, a second example of publication bias is to avoid the publication of negative studies. Melander *et al.* [48] compared 42 trials of five different selective seratonin re-uptake inhibitors submitted to the Swedish drug regulatory authority with 38 resulting publications. They found much selective and multiple publication of the same data. Of the 21 positive trials, 19 resulted in standalone publications, whereas of the 21 negative trials, only six were published as a standalone publication. Moreover, published pooled analyses of these trials were not comprehensive and failed to cross-reference each other.

These mechanisms of biasing both the results of individual trials and the total evidence base provided by trials are, of course, not an intrinsic limitation of randomised trials themselves. However the fact that the ideal randomised trial provides excellent evidence of treatment benefit is irrelevant if the quality of many real-world trials is compromised, thus limiting the ability to practice EBM. As noted above, there is an increasing momentum behind open science campaigns (for example, alltrials.net) to address these practical problems, through trial registries and through greater access to raw and unpublished data [14, 16–18].

### The problem of conducting the wrong trials

Industry and other interests influence the *way* trials are conducted and reported. Alongside this *which* trials get conducted is also affected by industry and other interests. In particular, trials are often conducted that ask questions that are not clinically important and waste resources [49]. For example, studies have demonstrated that the total output from randomised trials does not track the global burden of disease [50]. While this provides some indication that research priorities do not match global health problems, Chalmers *et al*. [49] note that is not the best or only way to capture the problem. For example, research agendas should also prioritise the burden caused by multi-morbidities, and should be sensitive to what is feasible and appropriate within a particular healthcare system.

Other studies have shown that randomised trials often investigate commercially but not clinically important questions. Industry interests favour potentially lucrative, patentable, treatments while neglecting rare diseases and treatments that are more difficult to exploit commercially [51]. Every-Palmer and Howick [52] illustrate this point by citing the lack of trials investigating exercise to treat depression, despite some existing evidence that it is of similar effectiveness to drug treatments. They suggest the benefits of exercise have ‘little commercial value because exercise cannot be patented’ [52]. Equally, industry interests do not just act to neglect less lucrative treatments, but also to widen the boundaries of diagnosis and expand existing markets, as well as turn social problems into medical conditions [51, 53].

Moreover randomised trials often investigate questions and measure outcomes that do not matter to patients and do not provide the evidence that clinicians need [54, 55]. In a letter to the Lancet, Liberati [56] discussed the 'avoidable uncertainties' that had persisted over 10 years of research into multiple myeloma. He cited the fact that of the 107 comparative phase 2 or phase 3 trials registered with clinicaltrials.gov only 58 had survival as an outcome, only 10 trials had it as a primary outcome, and no trials were head-to-head comparisons. In addition to industry interests, Liberati also blamed the general 'research governance strategy', noting for instance that researchers themselves often have conflicted interests and professional dis-incentives to perform head-to-head phase-three comparisons, and also that there are few explicit mechanisms for prioritising research.

More generally, issues of research prioritisation and 'agenda-setting' have been noted elsewhere [57]. Tallon *et al.* [54] compared the questions addressed in studies of treatments for osteoarthritis of the knee with the priorities and needs of 'research consumers' (rheumatologists, general practitioners, physiotherapists and patients). They found the literature was strongly focused on surgical and drug treatment, whereas patients and clinicians needed information and high-quality evidence about all treatment options. As in the examples given above by Every-Palmer and Howick, and Liberati, Tallon *et al*. suggest that this misalignment of priorities is due to industry funding bias and researchers’ conflicts of interest. They also list additional factors, including the lack of consumer research involvement in an agenda-setting. This latter issue, however, is one that has received extensive attention in recent years [58–60]. and many methods for involvement currently exist (for example, the James Lind Alliance Guidebook [61]).

### The problem of conducting the right trials the wrong way

Even where trials do align with clinically important questions, significant questions can still arise over how trials should be conducted and what constitutes methodologically appropriate design in a specific context. Typically, randomised trials are only undertaken when genuine uncertainty exists within the expert medical community as to the relative benefits of each intervention to be tested, a state known as equipoise [62]. This concept encapsulates a recurring dilemma faced in clinical research: how the scientific imperative to obtain more knowledge and improve the evidence base can be reconciled with the clinicians’ therapeutic duty to patients [63]. This dilemma was central to controversies over the use of randomised trials in research into AIDS treatment in the 1980s. Epstein [64, 65] showed how lay activist communities were supportive of the aims of trials seeking to develop new treatments, but were critical of trial methodologies that they saw as being unduly focused on generating ‘clean data’. Such fastidiousness sat uneasily with activists who were already incensed by drug regulation policies which they perceived as overly paternalistic, depriving them of the opportunity to assume the risks of trying experimental treatments [64]. Methodological demands for participants who had not previously taken other medication were viewed as discriminatory towards AIDS patients who had earlier sought to treat themselves [64]. Tensions between ‘fastidious’ trial design, which favoured homogeneity and the elimination of ambiguity, and ‘pragmatic’ designs that embraced the more messy, heterogenous aspects of clinical practice, were not new [66]. What they illustrate is that it may not always be possible, or desirable, to implement randomised trials on the basis of internal scientific validity alone. In the AIDS case, activists did win concessions in trial design around a more pragmatic approach to participation [64].

The AIDS trials case illustrates the enduring problem of the equipoise dilemma, in that judgements about the balance between scientific and therapeutic imperatives are necessarily imperfect and uncertain, particularly when such judgements become opened up to patient pressure. What can rightly be seen as methodological distortion when industry unduly biases the conduct and reporting of trials necessarily appears different when duty-of-care is at stake in cases where patients try to exert influence. This is not to say that the knowledge gained from randomised trials in such circumstances is necessarily less useful, but rather that randomised trials can be subject to significant, often inescapable, social pressures and professional dilemmas, which provide important contexts for their assessment as clinical evidence.

### Discussion – the social aspects of randomised trials

The limitations outlined above have implications for the development of advice and recommendations, for example, in the form of officially sanctioned guidelines such as those provided by the National Institute for Health and Care Excellence for treatments, screening programmes and other policy decisions. The efficacy of screening programmes (for example, for breast cancer) has been particularly controversial in recent years, with some experts arguing that the risks of over diagnosis in mammography are poorly understood and calling for an independent review of the evidence on benefits and harms of mammography (see exchange between Bewley [67] and Richards [68]). In this context, the UK National Screening Committee’s criteria highlight a need for evidence from high quality randomised trials that screening is effective in reducing mortality and morbidity. The largest-ever randomised controlled trial on outcomes from extension of mammographic screening from 50-70 years to 47-73 years is also underway [68].

Yet, such evidence will need to be put in the context of broader social and value-based questions on how we collectively engage with uncertain evidence, balance precaution and risk, and the distribution of rights and responsibilities that follow from new forms of knowledge. Sociologists have identified concerns about screening as a form of ‘surveillance’ and creation of new burdens on individuals (who are not ‘patients’) to conform to public health programmes, sensitivities in the process of gaining informed consent, and challenges people face in dealing with the necessarily uncertain knowledge produced by screening technologies [69, 70]. Equally, where access to screening is seen as an important benefit for health, similar questions to those raised in the AIDS case may arise when extension of breast cancer screening beyond the 50-70 years bracket is subject to randomisation. Healthcare professionals must also balance ambivalent evidence, delivery of care and cost pressures. Randomised trials cannot resolve these questions. Representing trials as a central part of EBM is, therefore, problematic as it strips away the more challenging aspects of the screening controversy. Indeed, the Screening Committee implicitly acknowledges this by adding a criterion that screening tests must be ‘clinically, socially and ethically acceptable to health professionals and the public’ (https://www.gov.uk/government/publications/evidence-reviewcriteria-national-screening-programmes/criteria-for-appraising-the-viability-effectiveness-andappropriateness-of-a-screening-programme). Qualitative research on different judgments that people make can inform this discussion on acceptability and also, desirability of specific interventions. The danger, though, is that trial evidence may crowd out such evidence by promising an impossible certainty of either a ‘positive’ (screening is effective) or ‘negative’ (there is no evidence that screening is effective) kind.

Historically, some commentators have highlighted the dangers of randomised trials unduly crowding out other forms of evidence in clinical settings [71]. However, the notion of ‘hierarchies’ of evidence within evidence-based medicine is no longer prevalent in the literature, being replaced by more nuanced typologies of evidence demonstrating how different research methods are appropriate for answering different types of research question [72, 73]. For example, Petticrew and Roberts [74] argue that randomised trials are most suited to questions of effectiveness, safety and cost effectiveness, but unsuited to addressing issues of salience, appropriateness, service delivery and service satisfaction. For these questions, qualitative research is found to be more appropriate. These social dimensions are critical; as Petticrew and Roberts point out, we have known for over 150 years that handwashing reduces infection, yet our knowledge of how to encourage increased handwashing remains poor. However, as we have shown above, the social dimensions of clinical practice are not confined to post-trial implementation of recommendations. The assumptions made within randomised trials themselves require interrogation. These may not just be limited to the dilemma of scientific and therapeutic concerns highlighted in the case of AIDS patient activism; they also stretch to issues of interpretation. As one psycho-oncologist commented regarding the independent review of breast screening:

- ‘The mantra that 'finding things early' is essentially a good thing is so inculcated into our collective psyche that even-handed appraisal of the data and rational decision-making is virtually impossible. I've worked within the field of breast cancer research for more than 27 years, have read all the opinions of epidemiologists and others, and scrutinised the latest publications, but even I remain uncertain about the value of screening mammography. I feel simultaneously silly for attending but scared not to do so’ [75].

Such self-reflection from experienced practitioners on the inbuilt assumptions within evidence architectures are vital, yet remain qualitative in nature and beyond the scope of quantitative analysis of randomised trials.

## Conclusions

In the end, randomised trials cannot substitute for expertise as is sometimes argued. Instead, the credibility of trial evidence can be enhanced by paying attention to the kinds of expertise required to make such evidence matter and by combining statistical knowledge with personal, experiential knowledge [76]. Evidence requires *interpretation* and never ‘speaks for itself’. That is, experts providing advice need to acknowledge different meanings and consider a *plurality* of sources and forms of evidence [77], and *institutions* play a key role in maintaining transparency and standards in both the production of evidence and its mediation by expert advisors [78]. These nuances risk being overlooked within a culture of standardisation that risks focusing on bureaucratic rules at the expense of patient-centred care [79, 80].

What Miller [81] describes as a ‘culture of reasoning’ within institutions, mediating different forms of evidence for decision-making purposes, will be important for the social value of randomised trials. To be sure, randomised trials can offer a counter-weight to unwarranted certainty or decision-making that rests on a narrow set of assumptions drawn from previous experience or personal bias. But judgments must still be made about the nature of the question a trial is meant to address (could it be asking the ‘wrong’ question?) and about the role of potential bias in interpreting the evidence generated (what assumptions have been made and could they be contested?). This is the paradox of randomised trial evidence: it opens up expert judgment to scrutiny, but this scrutiny in turn requires further expertise.

## Abbreviation

EBM: evidence-based medicine

## Footnotes

1 Thanks to Rachel Johnson for this example.
